# Prevalence of Bodily Distress Disorder Among a Sinhala Speaking Population Seeking Outpatient Treatment at a Tertiary Care Hospital, in Sri Lanka

**DOI:** 10.1192/bjo.2026.11099

**Published:** 2026-06-30

**Authors:** Amodha Medagedara, Haniska Anthony, JSU Fernando, Kolitha Jayasundara, KCD Mettananda, Ravini Premaratna, MSR Mallawa

**Affiliations:** 1 ELFT, Luton, United Kingdom; 2 Faculty of Medicine, University of Kelaniya, Ragama, Sri Lanka; 3 Colombo North Teaching Hospital, Ragama, Sri Lanka; 4 District General Hospital Negombo, Negombo, Sri Lanka; 5 Faculty of Medicine, University of Kelaniya, Ragama, Sri Lanka; (Foundation Year Doctor, National Hospital of Sri Lanka); (Foundation Year Doctor, Colombo North Teaching Hospital Sri Lanka)

## Abstract

**Aims:**

Bodily distress disorder (BDD) involves presence of excessively distressing somatic symptoms to which individuals direct excessive attention despite repeated contacts with healthcare providers or even if another condition is causing the symptoms,paying excessive out of proportion attention to symptoms. BDD is more prevalent following Covid-19, but data from Asia remains sparse. Thus, this study is done to determine the prevalence of BDD among patients seeking outpatient treatment at Colombo North Teaching Hospital (CNTH), Sri Lanka.

**Methods:**

We conducted a cross-sectional study recruiting consecutive, consenting patients attending the outpatient department (OPD) of CNTH from April to June 2025. Presence of somatic symptoms were determined by applying Bradford Somatic Inventory. Relationship of somatic symptoms to bodily distress disorder, depression or anxiety was determined according to ICD–11 criteria through clinical interviews by a senior registrar in psychiatry. In patients diagnosed with BDD lack of an adequate biological explanation for symptoms or in patients with established medical conditions contributing to symptoms, degree of attention to symptoms being excessive despite appropriate clinical examination, investigations and reassurance was confirmed by the Consultant Physician in charge of OPD.

**Results:**

We studied 236 patients (female 83.1%, mean age 62±11.8 years, educated up to orbelow grade eleven–83.1%, residency urban or suburban–89%). Prevalence of somatic symptoms was 94.1%. Out of those, BDD was diagnosed in 28.1%, depressive disorder in 14.3% and anxiety disorder in 7.1%. Prevalence of BDD was higher in females 29.1% compared with males 15% (Chi square 3.4,pvalue 0.06) and higher in people with education up to or below grade eleven–27.0% when compared with above grade eleven–25.0% (Chi square 0.7, p value 0.79). BDD was higher in people below 60 years (Chi square 5.6, p value 0.18) and in people residing in urban, and suburban areas (Chi square 11.0, p value 0.00). Number of comorbidities is positively correlated with BDD status. Most common two presenting symptoms were aches and pain (41.8%) and lack of energy/fatiguability (37.2%). Majority of those with BDD were on polypharmacy therapy (60.3%) and 22.2% were on analgesics.

**Conclusion:**

Prevalence of BDD among this whole sample of people seeking outpatient treatment at a tertiary care hospital in Sri Lanka was 26.7%. Age below 60years, urban and suburban residency,multi-morbidity were significantly associated with BDD.

